# Visual surveys provide baseline data on small vessel traffic and waterbirds in a coastal protected area

**DOI:** 10.1371/journal.pone.0283791

**Published:** 2023-04-13

**Authors:** Louise K. Blight, Douglas F. Bertram, Patrick D. O’Hara

**Affiliations:** 1 Procellaria Research & Consulting, Victoria, British Columbia, Canada; 2 School of Environmental Studies, University of Victoria, Victoria, British Columbia, Canada; 3 Wildlife Research Division, Environment and Climate Change Canada, Institute of Ocean Sciences, Sidney, British Columbia, Canada; 4 Canadian Wildlife Service, Pacific Wildlife Research Centre, Delta, British Columbia, Canada; 5 Department of Geography, University of Victoria, Victoria, British Columbia, Canada; MARE – Marine and Environmental Sciences Centre, PORTUGAL

## Abstract

The coastal waters of southern British Columbia, Canada, encompass habitat of international conservation significance to coastal and marine birds, including sizeable areas designated in the early 1900s as Migratory Bird Sanctuaries (MBS) to protect overwintering waterfowl from hunting near urban centres. Two of these, Shoal Harbour (SHMBS) and Victoria Harbour (VHMBS), have seen significant marine infrastructure development in recent decades and experience considerable vessel traffic. Vessel-related stressors are known to affect waterbirds, but traffic characteristics in coastal urban areas are poorly understood for the smaller vessels not tracked by Automatic Identification Systems (AIS). We conducted a pilot study using shore-based observers to develop small-vessel baselines for the winter months, when regional waterbird numbers are highest. During our surveys we recorded considerable inter-site variability in vessel traffic characteristics, with one site (SHMBS) a source of nearly twice as many vessel transits as the other (VHMBS). Most recorded vessels were small watercraft (mean length 26 ± 17′, mode 18′), and vessels at the high-traffic site were both shorter and faster on average. One in six vessels were classified as ‘noisy’, of interest given that noise is an important component of vessel disturbance of waterbirds and other marine animals. Few vessels (7% of all recorded) were of the type required to carry AIS transponders, which highlights the monitoring gap created by using AIS-based approaches alone in nearshore waters, and allows for correction of AIS-derived vessel counts. Waterbird community composition also varied by locality, with one site dominated by gulls (Laridae), cormorants (Phalacrocoracidae), and seaducks (Tribe Mergini), and the other by gulls, cormorants, and alcids (Alcidae). Our results demonstrate that fine-scale local variability must be taken into account when managing for vessel traffic disturbance of waterbirds, particularly at sites of high human population density and increasing coastal development.

## Introduction

Perhaps the best-known modern threat to marine and other waterbirds is acute marine oil pollution from shipping accidents, but additional shipping-related impacts to birds and marine systems are associated with commercial and recreational vessel traffic, including chronic oil pollution and various types of disturbance posed by vessel noise and presence [1–4]. Responses to disturbance by vessels can include sub-visible stress, flushing of nesting, foraging, or resting birds (and associated energetic impacts), exclusion from important habitat, or even death from collisions [3, 5]. Disturbance-related impacts can be short-term (stress and related physiological changes; loss of foraging opportunity) to longer-term (reduced body condition; seasonal exclusion), and can occur at the individual to population level [5, 6]. Different kinds of threats are posed by different vessel types and behaviours. These include vessel size, speed of travel, and angle of approach (e.g., escape response and collision are more likely with fast-moving and directly approaching motor craft; disturbance at nesting colonies or exclusion from foraging areas may be more likely with small vessels able to approach close to shore; cf. [7, 8]). Thus, understanding the characteristics of vessel traffic is vital to comprehending and mitigating disturbance responses by waterbirds, particularly in areas of seasonal importance to or occupation by a high number of individuals and/or use by at-risk species. Gaining an understanding of the scope and nature of modern threats in the marine environment is also an important component of updating protection offered by conservation reserves previously established under different baseline conditions.

**Fig 1:**
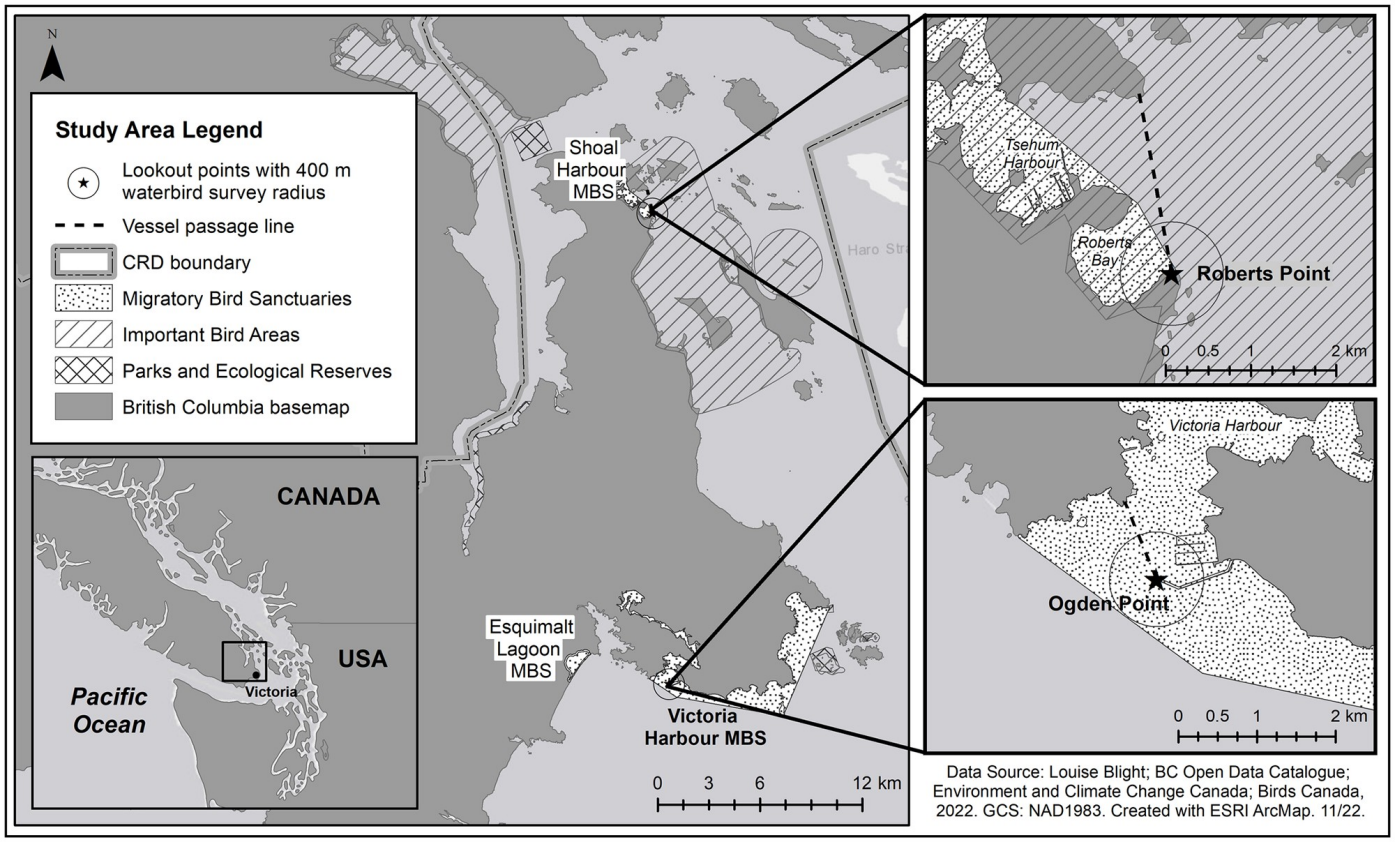
Study areas (Victoria Harbour and Shoal Harbour Migratory Bird Sanctuaries, Capital Regional District (CRD), Canada) showing spatial conservation designations, viewpoints, and passage lines (see text for details). Map generated by Alexandra King, Environment and Climate Change Canada. For the data source of Birds Canada (2022), listed in the figure, see [14].

The marine and nearshore areas of the Salish Sea, western North America’s largest inland sea, encompass areas of international conservation significance to marine birds and mammals [9–11]. On Canada’s Pacific coast, the city of Victoria and environs are home to three of the country’s 92 Migratory Bird Sanctuaries (MBS [12]), a designation under the federal *Migratory Birds Convention Act* (MBCA). The first three of these were established in the Gulf of Saint Lawrence in the province of Québec in 1919 and at Last Mountain Lake, Saskatchewan in 1921, followed by Victoria Harbour MBS in 1923—the first in Pacific Canada. Eight years later, in 1931, Shoal Harbour and Esquimalt Lagoon MBSs were designated in the Victoria region, reflecting Canada’s early recognition of these west coast marine sites as important habitat for waterbirds [12]. The 1,891 ha Victoria Harbour MBS fronts the municipalities of Esquimalt, Victoria, Oak Bay, and Saanich, while Shoal Harbour (146 ha) lies adjacent to the town of Sidney. Esquimalt Lagoon MBS (134 ha) is situated within the nearby municipality of Colwood (Fig 1). All of these communities form part of Greater Victoria, an area collectively governed as the Capital Regional District (CRD) and home to over 400,000 people [13].

Victoria’s MBSs were designated at a time when the primary threat posed to migratory waterbirds was commercial and recreational hunting. Indeed, the MBCA was brought into force in Canada in 1917 to protect birds from the steep population declines that had resulted from such overexploitation. Since its inception, MBS designation has meant the implementation of rules and prohibitions around taking, injuring, destroying, or molesting migratory birds, their nests, or their eggs in the sanctuaries. These restrictions include the prohibition of any hunting within MBS boundaries. While these statutes are still in place and remain powerful tools for bird protection, conservation measures within Greater Victoria’s working harbours have not kept pace with the region’s growth and development, reflecting the fact that marine conservation zones established near urban areas may provide poor protection against key threats [15]. Both Shoal Harbour and Victoria Harbour MBS have experienced significant development within their waters—something not explicitly restricted under MBS designation—and now are home to marinas, fuel docks, and other marine infrastructure. The southern portion of the Salish Sea also experiences among the highest volumes of vessel traffic in North America, including the recreational vessel traffic commensurate with a dense coastal population [16, 17].

Despite these potential sources of disturbance and pollution, these MBS still retain their importance to waterbirds nearly 100 years after they were first established (cf. [18, 19]), including areas used for winter foraging by at-risk species such as Ancient (*Synthliboramphus antiquus*) and Marbled Murrelet (*Brachyramphus marmoratus*) [20]. Though vessel disturbance to marine birds is an identified stressor in the Salish Sea [3], regional traffic patterns are not well understood, particularly for small vessels [21]. The advent of the marine Automatic Identification System (AIS) and the International Maritime Organization (IMO) requirement that AIS be fitted to international vessels of ≥ 300 gross tonnage, all cargo vessels of ≥ 500 gross tonnage, and to all passenger ships (at a minimum—national requirements vary, e.g. [22]) means access to automated monitoring data for a sizeable subset of shipping traffic worldwide. However, at the time of this study (2020–2021) most small non-commercial or non-passenger vessels in Canada, e.g., fishing vessels and pleasure craft, were not required to carry AIS transponders [22]. This means that for the purposes of disturbance studies, information on small vessel traffic behaviour and numbers is difficult to obtain as it must be estimated by means other than use of AIS data. This lack of information on small vessel traffic is a well-known problem in measuring vessel impacts on marine animals [21, 23, 24].

The purpose of our exploratory study was to initiate quantification of small vessel characteristics and movement patterns within two of these coastal protected areas, using approaches that can complement aerial and other remote survey methods already addressing similar questions across the broader region [21, 25, 26]. It is worth noting that Canada’s Gulf Island National Park Reserve, a 3600-ha complex of marine areas and small islands adjacent to our study area, aims to increase visitation and improve access to kayaks [27], something that could have negative impacts on certain coastal waterbird aggregations if ongoing disturbance occurs (cf. [28]). Our objective was to determine whether land-based surveys could be useful for determining local vessel traffic patterns in areas of importance for coastal waterbirds, with a secondary objective of gaining an understanding of temporal variability in vessel movement to inform future sampling efforts, and ground-truthing other, remote vessel monitoring results occurring in the region. This proof-of-concept work focused exclusively on characterizing marine vessel traffic in and around two MBS from late fall to early spring—the period of greatest waterbird abundance in the region [29, 30]. We thus also surveyed for waterbird presence, but we did not attempt to assess specific impacts by vessels on waterbirds in the study area given the preliminary nature of our research.

## Materials and methods

### Survey sites

Surveys took place at Victoria Harbour and Shoal Harbour MBS (Fig 1). Land-based sites have been successfully used as vantage points to determine coastal seabird movements and numbers in other jurisdictions [8, 31–33]. We used a similar approach to estimate vessel activity and waterbird presence in our study area, using a single viewpoint at each of our two study sites.

We chose our survey locations based on visibility from them of vessels exiting and entering each MBS, each of which circumscribes several harbours and marinas. For vessel observations at Shoal Harbour MBS (henceforth SHMBS), the public viewpoint at Roberts Point (48.662, -123.393, elevation ~2 m asl; Fig 1) was the only foreshore site with an unobstructed view of traffic entering the entire MBS area; SHMBS has a complex shoreline and encompasses Tsehum Harbour—which houses a fuel dock and about 12 marinas, boat yards, and yacht clubs—and Roberts Bay, which has several permanent mooring buoys in place.

For Victoria Harbour MBS (VHMBS), the only publicly-accessible viewpoint with a clear line of sight across the harbour entrance is the end of the Ogden Point breakwater (48.413, -123.393, elevation ~5 m asl; Fig 1). This breakwater protects the entrance to Victoria Harbour and its adjacent piers for cruise ships, Coast Guard vessels, and the marine pilotage. The viewpoint overlooks traffic to and from these and about six small-craft marinas, a fuel dock, a full-service shipyard, and other commercial operations, all located further within the harbour. It should be noted that although Victoria Harbour is the busiest sector of the VHMBS, being a hub for commercial vessels in the region as well as for recreational traffic, at > 1800 ha this MBS includes an extensive length of shoreline. It also encompasses two additional commercial marinas (Oak Bay Marina and Royal Victoria Yacht Club) in the adjacent municipality of Oak Bay, approximately 8 km away. These two marinas were not covered by our initial survey as there is not a single convenient viewpoint overlooking all three entrances. For budgetary reasons the present study excluded nearby Esquimalt Lagoon MBS.

Because surveys were carried out from publicly-accessible sites, and because no animals were manipulated or handled (waterbird records were observational only), no permits were required for this study.

### Vessel traffic observations

We conducted surveys simultaneously from the two viewpoints, with a single observer at each station. The same two observers were retained throughout the study, and surveyed the same site throughout it (with the exception of March 2020; see below). Depending on available daylight, surveys were conducted for 7 or 8 hours per day, with a 09:00 start. Surveys took place two days per month, one weekend (with ‘weekend’ including statutory holidays), and one weekday, from December 2020 to February 2021, for a total of 12 survey-days. Initial test surveys in March 2020 were to have taken place for the same number of days per site and for the same number of hours, but were curtailed by the COVID-19 pandemic and initial shelter-in-place public health orders. We instead conducted only a single survey at each site in March 2020—an initial test survey of 2.5 h at VHMBS, and a full 8-hour survey day at SHMBS, both by the same observer. We had originally intended to conduct each day’s surveys at a similar point in the tidal cycles, but the distance and complex coastal topography between these two sites resulted in temporally asynchronous cycles, with low tide at Sidney often 2–3 h later than low tide at Victoria, despite an overland distance of only ~30 km between the two locations. We instead focused on obtaining synchronized fine-scale vessel traffic data to gain an initial understanding of its temporal variability, which should facilitate appropriate stratification for future studies.

From each of the two viewpoints, we counted and characterized vessel traffic as it crossed a fixed ‘passage line’ [34] (Fig 1). We conceived of this as a linear boundary extending from the viewpoint to an opposing landmark near the entry to each site’s harbour, such that any vessel entering the MBS (Shoal Harbour) or Victoria Harbour proper (VHMBS) would have to cross it; the opposing landmark was approx. 1.4 km or 0.75 NM distant at Shoal Harbour, and 0.75 km or 0.4 NM at Ogden Point. Any vessel crossing this imaginary line was deemed to have entered or exited the MBS, and was recorded (see below). This simplified approach facilitated recording data on vessel movement and characteristics using only a single observer per site. Vessel observations were carried out using 8 × 42 binoculars and the naked eye. We did not attempt to track detailed vessel movements within each MBS. Nor did we track individual vessels; each vessel transit into or out of the MBS was recorded as a unique event.

For this study we defined “vessel” as any moveable floating craft propelled by human, engine, or sail power. We classified vessels based on propulsion type (sail, motor, motor-sail, paddle/row), use class (commercial, recreational, government, unknown—with ‘unknown’ being a vessel heard but not seen in poor visibility, i.e., brief periods of fog), and vessel type (canoe, kayak, row, stand-up paddle board, sailboat, inflatable, tug, tug & barge, pilot, fishing vessel, ferry, other motor craft, unknown). We also recorded estimated vessel speed (recording maximum and minimum speed to capture instances where vessels picked up or decreased speed when crossing the passage line, e.g., when departing or arriving at the harbours), length overall, and flag of country of registry or origin where present.

We estimated vessel speeds and lengths based on our experience with vessel operations, and on standardized visual cues, e.g., motor vessels traveling dead slow with little to no wake were estimated as travelling between ~3 and 7 knots; those that were not planing but were pushing a noticeable bow wave were estimated to be travelling at about 10 knots; those that were planing were recorded at their apparent speed. Individual names of larger commercial and public vessels (e.g., Canadian Coast Guard ships, commercial tugs, pilot vessels) were recorded and their travelling speeds and lengths overall obtained online. We used known length ranges of vessel brands, and on-deck objects such as dinghies, paddles, and people to help calibrate our estimates of vessel length, ‘Knots’ and ‘feet’ were used as the measurement units for estimated speed and length given our visual familiarity with these standard nautical measurements. As waterbirds may be sensitive to ambient sound [35, 36], we also classified vessels as ‘noisy’ or not, based on subjective impressions of vessel engine noise. ‘Noisy’ vessels were not simply those running motors, but, for example, were those operated at a volume that would impede normal conversation for people travelling on board.

### AIS assignation

To assess the minimum proportion of AIS-tracked vessels during our study, we assigned each observation to the category of a vessel being required by law to be fitted with an AIS (“AIS vessels”), or not (“non-AIS vessels”). At the time of the study, AIS technology was compulsory in Canada for international passenger vessels of ≥ 150 gross tonnage, other international vessels of ≥ 300 gross tonnage other than a fishing vessel, and vessels of ≥ 500 gross tonnage on domestic voyages. Passenger vessels certified to carry more than 12 passengers, or vessels ≥ 8 m in length and carrying passengers are also required to be fitted with an AIS (Class A or B) if on a voyage outside sheltered waters. Our study area is included in the regulations’ “sheltered waters" definition. However, additional requirements entered into force in April 2021, after the end of the field portion of our study: as well as the above-mentioned passenger vessels, AIS-A is now required on all vessels of ≥ 20 m (other than pleasure craft), dredges, those carrying dangerous goods or pollutants, and towboats ≥ 8 m in length [22, 37]. As we wanted to generate a measure of AIS coverage that would be relevant to future management, we assigned our vessel observations based on current (post-April 2021) requirements.

### Waterbird species

For the purposes of this study, waterbirds were defined as avian species using marine and intertidal environments exclusively or near-exclusively for a key component of their life history (e.g., foraging, aggregating, sheltering). This definition encompasses colonial and non-colonial waterfowl, loons and grebes, seabirds, and waders, but excludes members of the Passeriformes that forage in intertidal habitats along with many other habitat types (e.g., crows *Corvus* spp.). We also recorded raptors that forage on fish and/or waterbirds, in our area in winter Bald Eagle (*Haliaeetus leucocephalus*) and Peregrine Falcon (*Falco peregrinus*).

During vessel surveys, we recorded waterbird counts for 3 minutes each hour (December 2020 to February 2021), using 8 x 42 binoculars to scan within a 400 m radius and recording any birds present on the water or flying by. Birds unidentifiable to species level due to distance or weather conditions were identified to genus or family, and species identified by call alone were included. We used Google maps in advance of surveys to identify landmarks at 400 metres: at Victoria Harbour, this was the distance from the end of the Ogden Point breakwater to the tip of the second cruise ship pier, while for Shoal Harbour an exposed rock lies 400 m to the north of the Roberts Point viewpoint. As an index of waterbird use of each study area, we used our 3-minute point counts to generate both mean daily counts of all birds, and total number of individuals seen per species per site. While it is certainly possible that this latter value resulted in double-counting of some individuals, there is considerable waterbird movement through this region on an hourly and daily basis in the winter months (cf. [11]) and a total count of individuals observed over three minutes each hour was deemed the most useful way to summarize waterbird presence at our study sites. An incidental list was also recorded in eBird (‘incidental’ protocol [38]) for the duration of each vessel survey day, to assess the effectiveness of our 3-minute counts in capturing all species present over an entire day.

All data processing and analyses were carried out using R 4.0.0 [39] and packages car [40], dplyr, [41], and ggplot2 [42]. We checked assumptions of equal variance and normal distribution of data for each analysis, and adjusted statistical tests accordingly (i.e., Welch’s *t*-tests for our comparisons of two means; analysis of variance for comparing multiple means). Our Day 1 test survey (2.5 h, Ogden Point, VHMBS) was excluded from our analyses as it was a partial day only.

## Results

### Characteristics of vessel traffic

**Fig 2:**
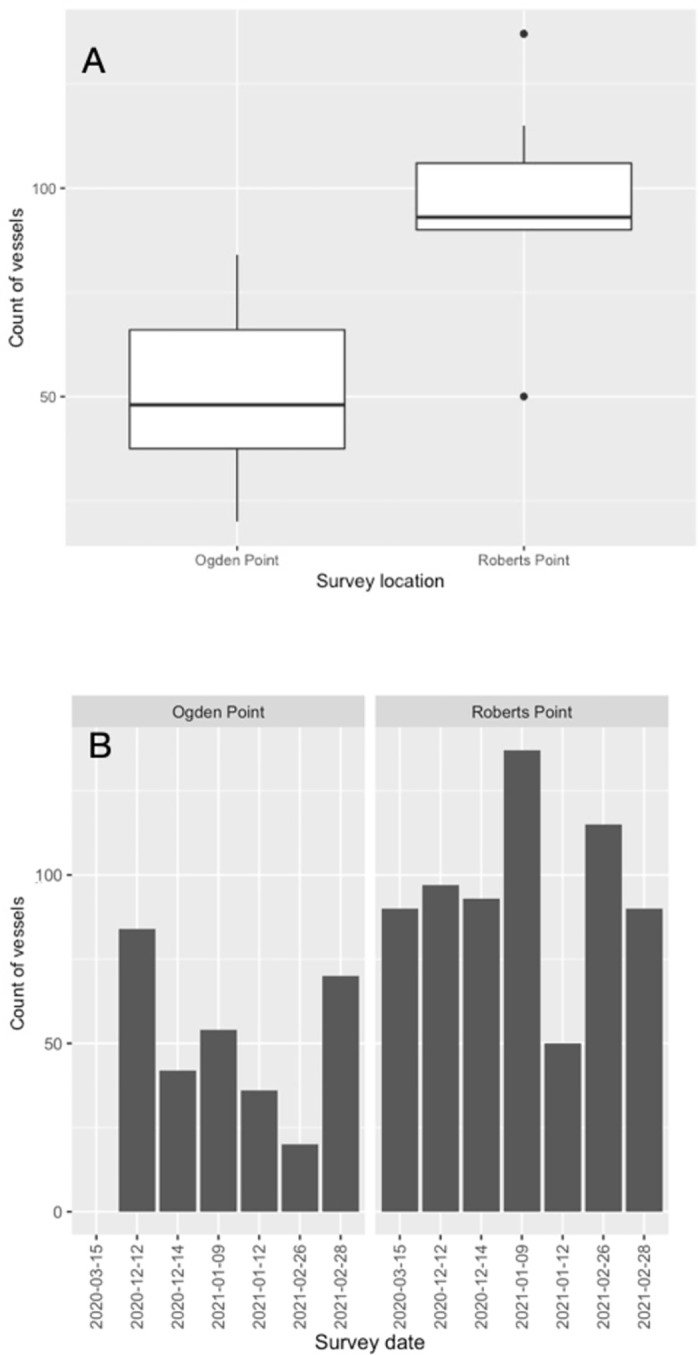
Boxplot summarizing daily counts of vessels (A) and raw daily count data (B) per survey site, Ogden Point (VHMBS), and Roberts Point (SHMBS), Greater Victoria, Canada.

We recorded a total of 978 vessel transits over our 13 full survey-days—about 75 vessel transits per day (± 33.6 (SD); max 191, min 86), or approximately 10 per hour on average over both survey locations. However, daily traffic volume varied between the two sites (*F* = 10.33, *p <* 0.01), with a daily maximum count at Roberts Point (SHMBS) of 137 vessels (min 50, mean 96 ± 26.6 (SD), *n* = 7), and of 84 vessels at Ogden Point (VHMBS; min 20, mean 51 ± 23.3 (SD), *n* = 6; Fig 2A and 2B). Total counts at Roberts Point over the three consecutive monthly counts (December 2020 to February 2021) were nearly double those of Ogden Point (582 vs. 306 vessels per site). The majority of vessels that we observed were classed by us as recreational (65% of all transits) or commercial (15%). This pattern was similar at each site with the majority of vessels classed as recreational at Ogden Point and Roberts Point (57% and 69%, respectively), and vessels in the ‘commercial’ category also the second-most numerous at Roberts Point (17%). However, at Ogden Point, recreational vessel counts were followed by government vessels (31% of total), a relatively high value driven by vessels transits from the nearby Pacific Pilotage Authority and the Canadian Coast Guard dock, as well as police and harbour patrol boats based in Victoria Harbour.

**Fig 3:**
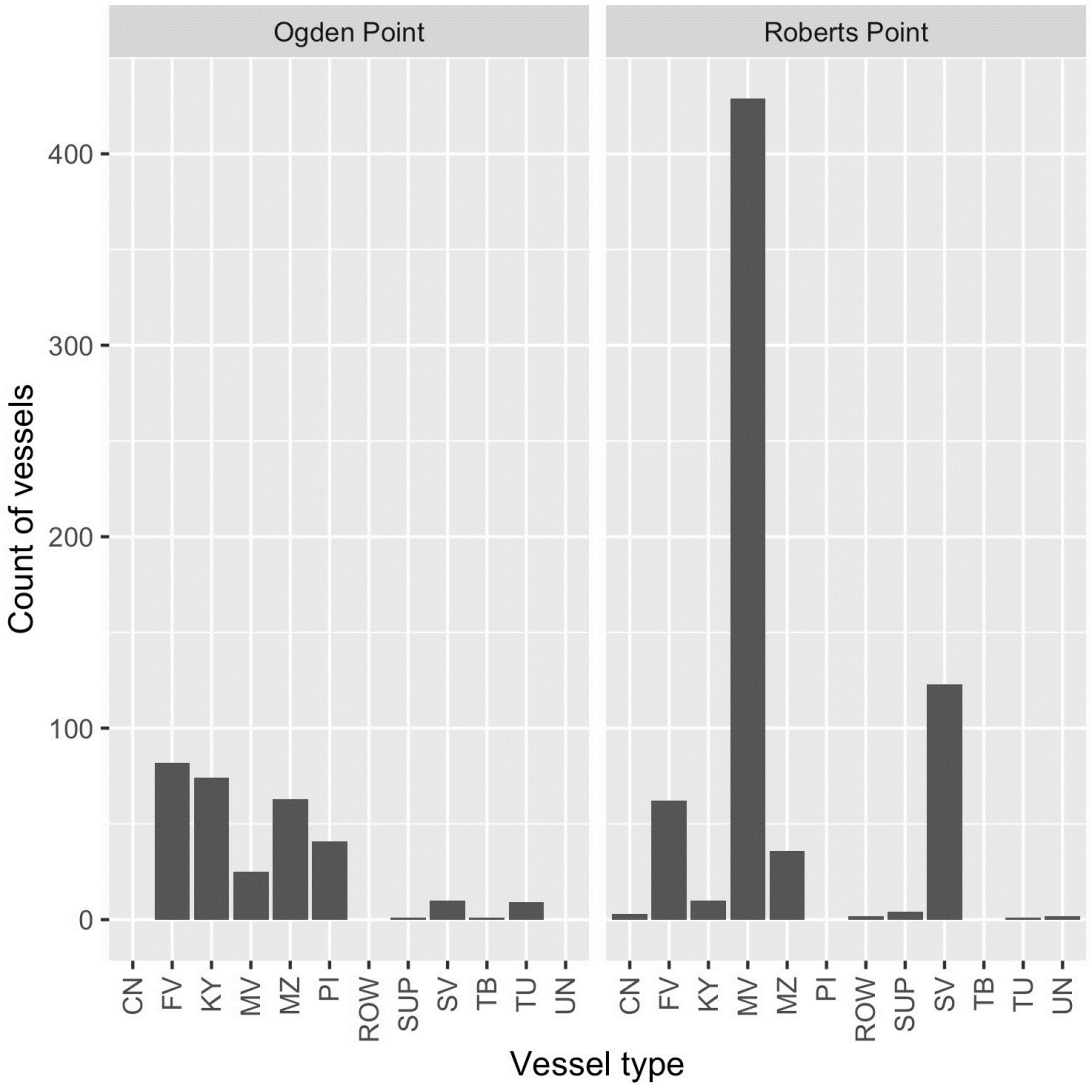
Vessel type count by survey site, Ogden Point (VHMBS) and Roberts Point (SHMBS), Greater Victoria, Canada. Abbreviations: CN = canoe, FV = fishing vessel, KY = kayak, MV = other motor vessel, MZ = inflatable, PI = pilot vessel, ROW = rowboat, SUP = stand-up paddle board, SV = sailboat, TB = tug and barge, TU = tug, UN = unknown (heard but not seen).

Most vessel traffic (77%; *n* = 751 transits) recorded from our two viewpoints was classified as one of various types of motor vessel (tug/tug and barge (*n* = 11), pilot vessel (*n* = 41), inflatable (*n* = 99), fishing vessel (*n* = 144), or other motor vessel (*n* = 456)), while 14% (*n* = 133) were sailboats, with the majority of these observed from Roberts Point, where other motor vessels and sailing vessels were disproportionately represented (Fig 3). Personal watercraft such as jet skis have featured prominently in other studies on vessel disturbance of waterbirds (e.g., [7]) but are not widely used in local waters and we did not record any. Discounting our ‘test’ survey day from Ogden Point, ferries were also absent during our study due to COVID-19 pandemic travel restrictions (see Discussion). Manually-powered craft such as kayaks and canoes made up the remainder (10%; *n* = 94; Fig 3) of all vessel transits recorded. Most of the kayaks we observed were in VHMBS, with 88% of all kayak transits recorded from Ogden Point; all pilot vessels occurred at this site (Fig 3).

**Fig 4:**
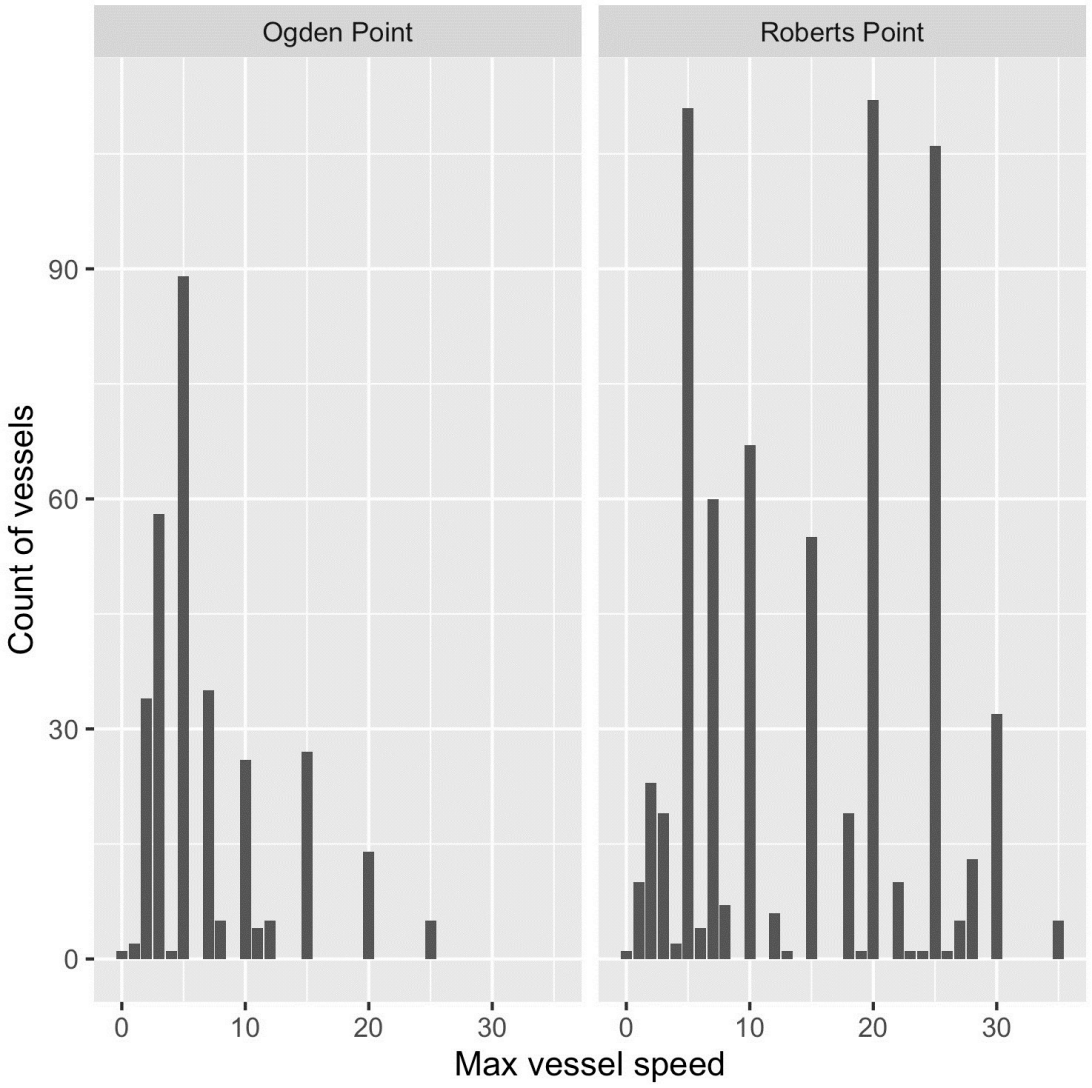
Frequency distribution of estimated maximum vessel speeds, Ogden Point (VHMBS) and Roberts Point (SHMBS), Greater Victoria, Canada.

**Fig 5:**
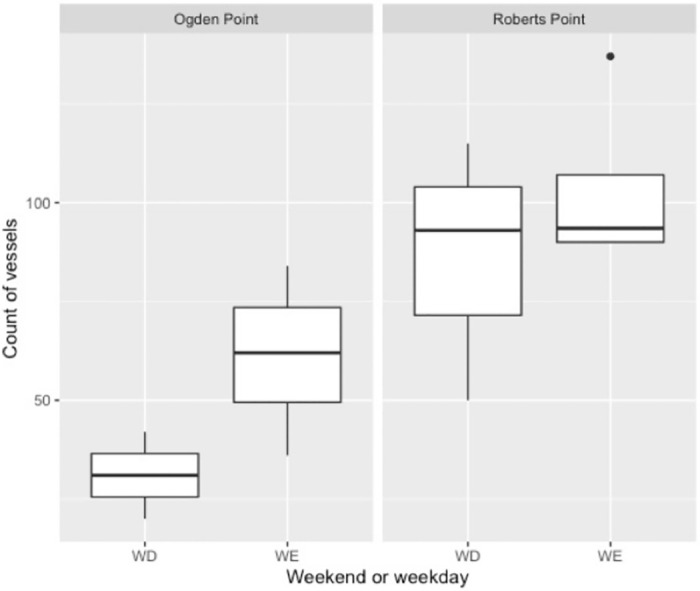
Distribution of daily vessel counts by day of week (weekend vs. weekday) and location, Ogden Point (VHMBS) and Roberts Point (SHMBS), Greater Victoria, Canada.

Vessel characteristics commensurately varied between the two survey locations. Mean estimated vessel length was 26 feet (± 17 (SD); mode 18 feet), but at Ogden Point, vessels were longer on average than those transiting Shoal Harbour (Roberts Point; Welch’s *t*-test, *t* = 2.61, *p <* 0.01). The estimated speeds at which vessels transited passage lines ranged from 0 (drifting vessels) to 35 knots. Mean estimated speed was 11 knots (± 9 (SD); mean minimum speed) to 12 knots (± 9; mean maximum speed), but vessels observed from Roberts Point were faster on average than those at Ogden Point (based on maximum estimated speed; Welch’s *t*-test, *t* = -17.03, p < 0.001, Fig 4). The number of vessel transits trended toward being higher on weekend days than on weekdays but this difference was not significant (*F* = 2.90, *p* = 0.12; Fig 5). Transits by vessels that were subjectively classified as “noisy” (e.g., high-speed pilot vessels; fast pleasure or commercial craft with high-horsepower outboard motors) made up 16% (*n* = 153) of those recorded. The only US-flagged vessels we recorded were on 15 March 2020, after the global COVID-19 pandemic was declared but prior to international travel restrictions that were put in place at the end of that month and extended into the autumn of 2021.

### AIS

Only 72 vessel transits, or 7% of all observations, were by vessels of the tonnage or class requiring that AIS be fitted. This represents a minimum number of AIS vessels as vessel operators may install AIS transponders despite no legal requirement to do so, and voluntary uptake of AIS has increased in the region over time [21]. Our approach of visual observations only meant that we were unable to determine which vessels actually carried AIS transponders.

### Waterbird species

**Fig 6:**
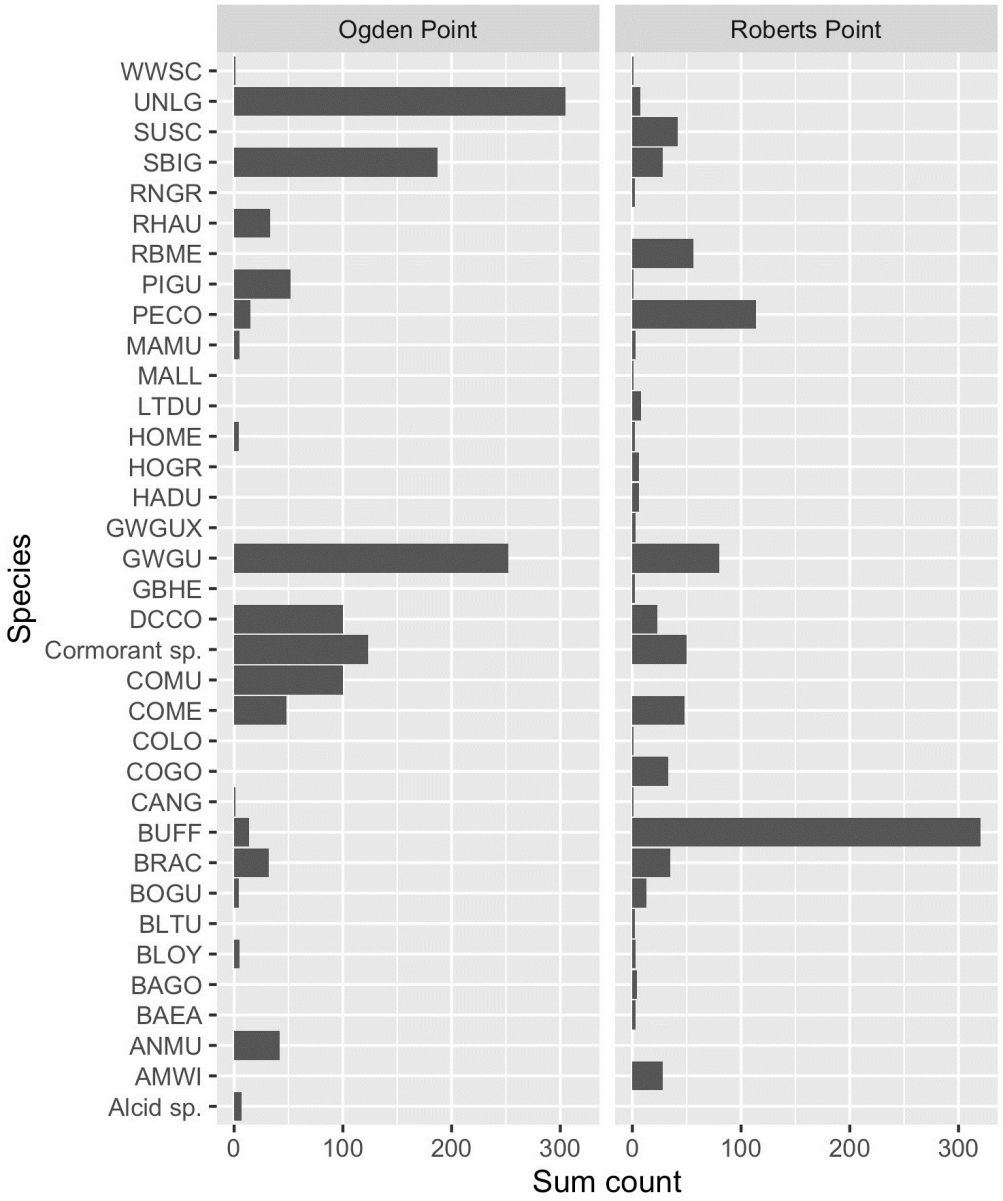
Total counts of waterbird species recorded on hourly 3-minute surveys over 12 survey days, December 2020 to February 2021, Ogden Point (VHMBS) and Roberts Point (SHMBS), Greater Victoria, Canada. Abbreviations are standardized 4-letter alpha codes: AMWI = American Wigeon (*Mareca americana*); ANMU = Ancient Murrelet (*Synthliboramphus antiquus*); BAEA = Bald Eagle (*Haliaeetus leucocephalus*); BAGO = Barrow’s Goldeneye (*Bucephala islandica*); BLOY = Black Oystercatcher (*Haematopus bachmani*); BLTU = Black Turnstone (*Arenaria melanocephala*); BOGU = Bonaparte’s Gull (*Chroicocephalus philadelphia*); BRAC = Brandt’s Cormorant (*Urile penicillatus*); BUFF = Bufflehead (*Bucephala albeola*); CANG = Canada Goose (*Branta canadensis*); COGO = Common Goldeneye (*Bucephala clangula*); COLO = Common Loon (*Gavia immer*); COME = Common Merganser (*Mergus merganser*); Cormorant sp. = Cormorant species (Phalacrocoracidae); DCCO = Double-crested Cormorant (*Nannopterum auritum*); GBHE = Great Blue Heron (*Ardea herodias*); GWGU = Glaucous-winged Gull (*Larus glaucescens*); GWGUX = Glaucous-winged Gull hybrid; HADU = Harlequin Duck (*Histrionicus histrionicus*); HOGR = Horned Grebe (*Podiceps auritus*); HOME = Hooded Merganser (*Lophodytes cucullatus*); LTDU = Long-tailed Duck (*Clangula hyemalis*); MALL = Mallard (*Anas platyrhnychos*); MAMU = Marbled Murrelet (*Brachyramphus marmoratus*); PECO = Pelagic Cormorant (*Urile pelagicus*); PIGU = Pigeon Guillemot (*Cepphus columba*); RBME = Red-breasted Merganser (*Mergus serrator*); RHAU = Rhinoceros Auklet (*Cerorhinca monocerata*); RNGR = Red-necked Grebe (*Podiceps grisegena*); SBIG = Short-billed Gull (*Larus brachyrhynchus*); SUSC = Surf Scoter (*Melanitta perspicillata*); UNLG = Unidentified *Larus* gull; WWSC = White-winged Scoter (*Melanitta deglandi*).

At Roberts Point (SHMBS), our 3-minute point counts recorded a total of 31 species or species groups (e.g., “cormorant sp.”) over 6 survey days in 2020–2021, and 21 at Ogden Point (VHMBS), for a total of 36 taxa at both sites combined. Bufflehead (*Bucephala albeola*), gulls (Laridae), and cormorants (Phalacrocoracidae) were the most numerous species or species groups observed overall (Fig 6). After gulls and cormorants, alcids (Alcidae; particularly Common Murre *Uria aalge*) were the next most common taxonomic group at Ogden Point, but few alcids were recorded at Roberts Point, where seaducks (Tribe Mergini) predominated (Fig 6). While Ogden Point was less species-rich, the number of individuals using the site was higher (mean of daily point count totals 199 ± 55 SD) than at Roberts Point (154 ± 27 SD). Our ‘incidental’ lists indicated that the 3-minute point counts captured from 67–93% of the species present on a given survey day; 8 species were missed entirely by the 3-minute counts, with all of these being species observed on a single occasion. Although having only a single observer at each site meant we were unable to record individual waterbird behaviours in response to vessel traffic, we did note that birds generally tended to show an obvious disturbance response (diving or flying away) only when vessels moved directly toward them, and that the same responses were elicited by vessels of different types, from kayaks to fast motorized craft.

## Discussion

Our shore-based approach to recording marine traffic allowed for detailed, non-automated characterization of small and mid-sized vessels using two coastal areas of importance to coastal marine birds. Results were necessarily tentative given the preliminary nature of this study (13 days over 4 months), particularly as initial survey efforts were cut short by early COVID-19 pandemic lockdown measures in mid-March 2020. Nonetheless, our results provide a proof-of-concept for shore-based vessel surveys that are applicable to the design of future studies, and an initial characterization of local traffic patterns. Despite vessel traffic volume in the region being known to be much lower in winter months than in summer [21, 23, 25], we recorded relatively high local vessel counts, including a maximum count of 137 transits over a 7-hour day at Roberts Point. Despite the geographical proximity of the two MBS, vessel traffic volume and characteristics varied considerably by survey site—with one site (Ogden Point, VHMBS) represented by fewer, larger and slower vessels on average (and with the majority of kayaks observed in the study; Fig 3), and the other (Roberts Point, SHMBS) by numerous smaller and generally faster transits—presumably reflecting the different infrastructure and facilities found at the two harbours, Although the difference was non-significant, vessel traffic volume also trended toward being heavier on weekends, a pattern that has also been observed regionally, with the difference disproportionately greater during winter vs. summer [26].

At both survey sites, mean vessel speed exceeded the 5-knot speed limit typically posted in inner harbour waters. Though our hypothetical passage lines (the point at which vessel data were recorded) were situated just outside the 5-knot speed zones for each site, the nearly-identical mean values for estimated minimum and maximum vessel speeds (11 vs. 12 knots) means that most vessels were travelling at a constant speed as they crossed the passage line, i.e., operators were generally not slowing down on approach to the harbour entrances, or speeding up as they exited them. Because marine bird disturbance responses are known to increase with vessel speed [7, 43], additional speed limits within MBS boundaries and increased enforcement throughout may be appropriate tools to consider as a management option within coastal MBSs, or within any sub-areas of these used by aggregations of waterbirds.

The relative proportion of vessel types that we observed were broadly similar to those recorded during another study conducting aerial surveys of the entire Salish Sea over several months, where 42%, 19%, and 12% of vessels were motorized pleasure craft, sailboats, and fishing vessels, respectively [21] (vs. 47% (“other motor vessel”), 14%, and 15%, this study). Interestingly, a full ten percent of the traffic we observed was comprised of non-motorized craft (kayaks, stand-up paddle boards, rowboats, canoes). This is perhaps reflective of the inshore nature of our surveys, but may be relevant for management considerations given the potential for kayaks to disturb nesting or roosting seabirds and foraging benthic feeders like seaducks [28, 44], perhaps resulting from their ability to move close to shore with no warning of their approach. In contrast, about 1 in 6 of all vessel transits, or 16%, were identified as being ‘noisy’. Although our classification of vessels in this category was a subjective one, this preliminary result is worth consideration given that noise is a specific component of vessel disturbance in aquatic systems [45], and marine birds may respond to noise as a disturbance [35, 36, 46]. While effects of vessel noise alone has so far not been well studied in the context of waterbird responses [3], it has been explicitly described as a supposed component of overall vessel disturbance effects (e.g., [7, 47, 48]). We identified vessels as noisy based on apparent in-air noise, but some vessels will contribute to both in-air and underwater noise (with the latter unmeasured by us in this study). Effects of underwater noise on birds are perhaps even more poorly understood than are those of in-air noise [46, 49], but are likely have both direct and indirect consequences.

Unsurprisingly, we identified vessel traffic patterns reflective of the international COVID-19 pandemic controls in place during the time of our field surveys. With the exception of the single Shoal Harbour survey on 15 March 2020 (a date that was after the pandemic had been declared by the World Health Organization on 11 March, but before travel restrictions were imposed), we recorded no vessels with US flags, and no ferries. The only regular passenger ferries that operate in our survey area during winter are those travelling between the US and Canada, out of Victoria Harbour; these services were suspended over the course of our surveys due to pandemic-related border closures from the end of March 2020 to early September 2021 (or later in the case of some operations). Measurable changes in maritime traffic since the start of the COVID-19 pandemic, particularly during lockdowns, have been documented in other waters worldwide [50–52]. Though we lack pre-pandemic observations to serve as controls, local waters and MBS would by definition see higher numbers of transits by non-Canadian vessels outside of times of international travel restrictions; whether this decrease in international vessel transits was offset to any degree by a pandemic-related, post-lockdown increase in local traffic, as has been seen elsewhere [50], is currently unknown.

Our observations allowed us to estimate the minimum proportion of non-AIS vessel transits in our study area, but because this estimate was solely based on observed vessel type (i.e., whether required by law to carry an AIS transponder), it is an overestimate of non-AIS vessels to some unknown degree. Only 7% of the transits we observed were by a vessel class required to have AIS installed as of 27 April 2021, the date of a regulatory change. However, other studies in the region have used remote survey techniques (aerial surveys or automated cameras in conjunction with AIS) and estimated the proportion of small vessels not using AIS (and not required to do so); these estimates range from 85–90% (for sailboats, pleasure craft, and fishing vessels [21]) to 67–96% (fishing vessels, ecotourism vessels, sailboats, and motor craft [26])—meaning up to 33% of AIS-exempt vessels are operating an AIS.

Because our surveys were focused on vessel traffic characteristics, we did not record details of any waterbird disturbance caused by vessels. However, for SHMBS we did observe that birds on the water were more likely to flush when motor vessels deviated from the transit routes to and from the main harbour entrance, suggesting either that birds using, e.g., nearshore areas, are selecting foraging or resting sites where they anticipate no disturbance, or alternatively, that birds are habituated to daily vessel behaviours, but not to deviations from those. Burger [7] similarly found that waterbirds at a colony responded more strongly to vessels travelling outside an established channel; any increase to vessel traffic on weekends (see above) has implications under the latter scenario. Our sub-sampling approach to recording waterbird presence generally worked well, with 3-minute point counts recording from 67–93% of all species present on a given survey day; 8 species recorded throughout the day using the eBird ‘incidental’ protocol were missed entirely by these 3-minute counts. Species recorded only once, as single individuals or in low numbers, were most likely to be missed by this approach. Point counts were also adequate to broadly characterize the similarities and differences among the guilds using the two sites in winter: the vicinity of VHMBS, on the edge of the open water of the Strait of Juan de Fuca, was used more heavily by alcids, while the enclosed embayments near SHMBS saw higher use by seaducks. Gulls and cormorants were common at both sites, though more numerous at Ogden Point. For future studies we recommend two observers per site if waterbird behaviours are to be recorded along with vessel traffic characteristics; assessing such behaviours will also require measurements of vessel distances and travel direction relative to birds [8, 43]. Day-long ‘incidental’ observations are required where it is important to record all bird species occurring at a study site.

## Conclusions

While vessel-related impacts from oil pollution have long been known to affect marine birds (e.g., [53, 54]), understanding the disturbance impacts of marine vessel traffic is still a work in progress, particularly given the species- and situation-specific responses of waterbirds to the various types of vessel disturbance [2, 43, 55]. Characterizing vessel traffic, including the component formed by non-AIS vessels, is therefore key to understanding and mitigating disturbance responses by waterbirds. Our proof-of-concept study provides an approach to understanding fine-scale patterns in local environments, and one that can be used to ground-truth the remote data collection efforts (e.g., using historical and real-time AIS data, or the use of satellite optical sensor imagery such as Google Earth images to estimate traffic [26, 56]) required over wider geographic areas. This approach could also be used to collect a baseline for small vessel traffic in areas of interest for marine conservation planning, something that has been identified as necessary in studies using AIS and other remote approaches to characterize vessel traffic over wider areas [25]. This approach would also contribute to planning for species that require areas of little or no disturbance [57], and may be relevant to understanding disturbance threats to waterbirds nesting at island colonies in the region given that approaches by vessel traffic can also disturb nesting and roosting marine birds [5, 7, 28]. Although budget constraints and pandemic restrictions limited the scope of our study to a set of snapshots in time, it nonetheless provided a preliminary set of fine-scale observations that will allow for improved quantification or measures of the timing and nature of local disturbances to waterbirds from vessel traffic. The inter-site differences in our results—despite the superficial similarity of the two sites and their geographical proximity—suggest that future studies carrying out more comprehensive assessments of local vessel traffic will need to take into account this sort of refined spatial and temporal variability, and stratify field studies accordingly.

Whether our survey data spanning four winter months are representative of future marine traffic conditions in the region remains to be discovered through more detailed work. Regardless, as the regional human population grows and vessel traffic increases with it [13, 25], it will be increasingly important to understand the pressure points of human impacts on marine wildlife populations existing at the interface of urban and natural areas. Understanding the patterns and numbers of vessel traffic in and around urban Migratory Bird Sanctuaries and other conservation areas is a vital part of ensuring that areas set aside for waterbirds long ago can continue to provide habitat for them into the future.

## References

[1] Burger AE, Fry DM. 1993. Effects of oil pollution on seabirds in the northeast Pacific. In Vermeer K, Briggs KT, Morgan KH, Siegel-Causey D, editors. The status, ecology, and conservation of marine birds of the North Pacific. Ottawa: Canadian Wildlife Service Special Publication; 1993. pp. 254–263.

[2] Schwemmer P, Mendel B, Sonntag N, Dierschke V, Garthe S. Effects of ship traffic on seabirds in offshore waters: implications for marine conservation and spatial planning. Ecol Appl. 2011;21(5):1851–60. doi: 10.1890/10-0615.1 21830723

[3] Hentze N. The effects of marine vessel traffic on marine birds in British Columbia: A literature review. Technical Report Series No. 2020. Delta (BC): Canadian Wildlife Service, Pacific and Yukon Region; 2020. ix + 90 pp.

[4] Carreño A, Lloret J. Environmental impacts of increasing leisure boating activity in Mediterranean coastal waters. Ocean Coast Manag. 2021;209:105693.

[5] Carney KM, Sydeman WJ. A review of human disturbance effects on nesting colonial waterbirds. Waterbirds. 1999;22:68–79.

[6] Frid A, Dill LM. 2002. Human-caused disturbance stimuli as a form of predation risk. Conserv Ecol. 2002;6(1):11.

[7] Burger J. 1998. Effects of motorboats and personal watercraft on flight behavior over a colony of Common Terns. Condor. 1998;100(3):528–534.

[8] Ronconi RA, Clair CC. Management options to reduce boat disturbance on foraging black guillemots (*Cepphus grylle*) in the Bay of Fundy. Biol Conserv. 2002;108(3):265–71.

[9] Butler RW. Twenty years on: advances in ecological understanding of globally important birds in the Strait of Georgia, British Columbia, and Puget Sound, Washington. Mar Ornithol. 2009;37:1–2.

[10] Bertram D.F. 2014. The coastal birds. In: Beamish R, McFarlane G, editors. The sea among us: The amazing Strait of Georgia. Nanaimo, BC: Harbour Publishing; 2014. pp. 211–262.

[11] Butler RW, Couturier AR, Bradley DB, editors. The Salish Sea marine bird and mammal atlas. Delta and Port Moody (BC): Birds Canada and Pacific Wildlife Foundation; 2021. https://storymaps.arcgis.com/stories/643e7710d56a427487e4fbe04cb8064c

[12] Government of Canada [internet]. Migratory bird sanctuaries across Canada. 2021 [cited 19 January 2022]. https://www.canada.ca/en/environment-climate-change/services/migratory-bird-sanctuaries/locations.html

[13] Capital Regional District (CRD). Demographics: Population estimates, July 1st Capital Region [internet]. Victoria (BC): Capital Regional District; 2022 [cited 15 August 2022]. https://www.crd.bc.ca/docs/default-source/regional-planning-pdf/population/population-pdfs/2021_populationestimate.pdf?sfvrsn=f9c7e3cd_0

[14] Birds Canada. Important Bird and Biodiversity Areas in Canada. 2022 [cited 23 November 2022] https://www.ibacanada.org

[15] Boersma PD, Parrish JK. Limiting abuse: marine protected areas, a limited solution. Ecol Econ. 1999;31:287–304.

[16] Gray DL, Canessa RR, Keller CP, Dearden P, Rollins RB. Spatial characterization of marine recreational boating: Exploring the use of an on-the-water questionnaire for a case study in the Pacific Northwest. Mar Policy. 2011;35(3):286–98.

[17] Simard Y, Roy N, Giard S, Yayla M. Canadian year-round shipping traffic atlas for 2013: Volume 3, West Coast. Can Tech Rep Fish Aquat Sci. 2014;3091(Vol.3)E.

[18] Capital Regional District (CRD). Migratory Bird Sanctuaries of the Capital Region [internet]. Victoria (BC): Environmental Protection, Capital Regional District; 2016 [cited 15 August 2022]. https://www.crd.bc.ca/docs/default-source/es-harbours-pdf/bird-santuary/migratorybirdsanctuaryouter-final.pdf?sfvrsn=2.

[19] Smart A. Victoria Harbour’s rebirth is for the birds: Wildlife populations returning as a result of cleanup campaigns in recent decades. Victoria Times-Colonist. 2017 April 23 [cited 15 August 2022]. https://www.timescolonist.com/islander/victoria-harbour-s-rebirth-is-for-the-birds-1.16551964.

[20] Holm KJ, Burger AE. Foraging behavior and resource partitioning by diving birds during winter in areas of strong tidal currents. Waterbirds. 2002;25(3):312–325.

[21] Serra-Sogas N, O’Hara PD, Pearce K, Smallshaw L, Canessa R. Using aerial surveys to fill gaps in AIS vessel traffic data to inform threat assessments, vessel management and planning. Mar Policy. 2021;133:104765.

[22] Government of Canada [internet]. Navigation Safety Regulations, 2020. Current to 2022-01-24 [cited 12 February 2022]. https://laws-lois.justice.gc.ca/eng/regulations/SOR-2020-216/FullText.html.

[23] MacGillivray A, Wood M, Li Z, Allen A, Hannay D. Regional ocean noise contributors analysis: enhancing cetacean habitat and observation program. Victoria (BC): JASCO Applied Sciences; 2016 Document 01195, Version 3.0. Technical report for Vancouver Fraser Port Authority.

[24] Merchant ND, Brookes KL, Faulkner RC, Bicknell AW, Godley BJ, Witt MJ. Underwater noise levels in UK waters. Sci Rep. 2016;6(1):1–0. 27830837 10.1038/srep36942 PMC5103265

[25] McWhinnie LH, O’Hara PD, Hilliard C, Le Baron N, Smallshaw L, Pelot R et al. Assessing vessel traffic in the Salish Sea using satellite AIS: An important contribution for planning, management and conservation in southern resident killer whale critical habitat. Ocean Coast Manag. 2021;200:105479.

[26] O’Hara PD, Serra-Sogas N, McWhinnie L, Pearce K, Le Baron N, O’Hagan G et al. Automated identification system for ships data as a proxy for marine vessel related stressors. Sci Total Environ. 2023;865:160987. doi: 10.1016/j.scitotenv.2022.160987 36563755

[27] Parks Canada [internet]. Gulf Islands National Park Reserve: Get involved! Developing the Management Plan, 2018 [cited 28 March 2021]. https://www.pc.gc.ca/en/pn-np/bc/gulf/plan.

[28] Chatwin TA, Joy R, Burger AE. Set-back distances to protect nesting and roosting seabirds off Vancouver Island from boat disturbance. Waterbirds 2013;36: 43–52.

[29] Campbell EC, Campbell RW, McLaughlin RT. Waterbirds of the Strait of Georgia. Vancouver: MacMillan Bloedel Limited and British Columbia Waterfowl Society; 1991.

[30] Pearson SF, Barry K, Davidson P, Evenson J, Raphael MG, Ross T, et al. 2014. Status and trends of the Salish Sea’s marine birds. Salish Sea Ecosystem Conference [Abstract]. Seattle, Washington, 1 May 2014 [cited 19 January 2022]. https://cedar.wwu.edu/ssec/2014ssec/Day2/67/.

[31] Sullivan TM, Butler RW, Boyd WS. Seasonal distribution of waterbirds in relation to spawning Pacific herring, *Clupea pallasi*, in the Strait of Georgia, British Columbia. Can Field Nat. 2003;116(3):366–70.

[32] Elmberg J, Hirschfield E, Cardoso H. Diurnal seabird movements at Cabo Carvoeiro (Peniche, Portugal): observations in early October 2012. Seabird. 2013;26:24–30.

[33] Elmberg J, Hirschfield E, Cardoso H, Hessel R. Passage patterns of seabirds in October at Cabo Carvoeiro, Portugal, with special reference to the Balearic Shearwater *Puffinus mauretanicus*. Mar Ornithol. 2016;44:151–156.

[34] Nuka Research and Planning. West Coast Spill Response Study. Volume 2: Vessel Traffic Study. Plymouth (MA) and Seldovia (AK): Nuka Research and Planning Group; 2013. Report to the BC Ministry of Environment.

[35] Buxton RT, Galvan R, McKenna MF, White CL, Seher V. Visitor noise at a nesting colony alters the behavior of a coastal seabird. Mar Ecol Prog Ser. 2017;570:233–46.

[36] Pichegru L, Vibert L, Thiebault A, Charrier I, Stander N, Ludynia K, et al. Maritime traffic trends around the southern tip of Africa–Did marine noise pollution contribute to the local penguins’ collapse? Sci Total Environ. 2022; 849:157878. doi: 10.1016/j.scitotenv.2022.157878 35944629

[37] Government of Canada [internet]. New Navigation Safety Regulations. Ship Safety Bulletin 23/2020, RDIMS No. 16889859, Transport Canada [cited 12 February 2022]. https://tc.canada.ca/sites/default/files/2021-05/SSB-23-2020E.pdf.

[38] eBird. Guide to eBird protocols: Incidental. eBird, Cornell Lab of Ornithology, Ithaca, New York; 2021 [cited 15 January 2023]. https://support.ebird.org/en/support/solutions/articles/48000950859-guide-to-ebird-protocols#anchorIncidental.

[39] R Core Team. R: A language and environment for statistical computing. Vienna: R Foundation for Statistical Computing; 2020. https://www.R-project.org/.

[40] Fox J, Weisberg S. An R companion to applied regression. 3rd Ed. Thousand Oaks (CA): Sage; 2019.

[41] Wickham H, François R, Henry L, Müller K. dplyr: A grammar of data manipulation. R package version 1.0.10; 2022 Sept 01. https://CRAN.R-project.org/package=dplyr.

[42] Wickham H. ggplot2: Elegant graphics for data analysis. New York: Springer-Verlag New York; 2016.

[43] Bellefleur D, Lee P, Ronconi RA. The impact of recreational boat traffic on Marbled Murrelets (*Brachyramphus marmoratus*). J Environ Manage. 2009;90(1):531–8. 18222029 10.1016/j.jenvman.2007.12.002

[44] Carter HR, Hébert PN, Clarkson PV. Decline of Pelagic Cormorants in Barkley Sound, British Columbia. Wildl Afield. 2007;4:4–32.

[45] Williams R, Wright AJ, Ashe E, Blight LK, Bruintjes R, Canessa R, et al. Impacts of anthropogenic noise on marine life: Publication patterns, new discoveries, and future directions in research and management. Ocean Coast Manag. 2015;115:17–24.

[46] Hansen KA, Hernandez A, Mooney TA, Rasmussen MH, Sørensen K, Wahlberg M. The common murre (*Uria aalge*), an auk seabird, reacts to underwater sound. J Acoust Soc Am. 2020;147(6):4069–74. 32611143 10.1121/10.0001400

[47] Rodgers JA Jr, Schwikert ST. Buffer-zone distances to protect foraging and loafing waterbirds from disturbance by personal watercraft and outboard-powered boats. Conserv Biol. 2002;16(1):216–224. doi: 10.1046/j.1523-1739.2002.00316.x 35701971

[48] Rodgers JA Jr, Schwikert ST. 2003. Buffer zone distances to protect foraging and loafing waterbirds from disturbance by airboats in Florida. Waterbirds. 2003;26(4):437–443. 10.1046/j.1523-1739.2002.00316.x 35701971

[49] Dooling RJ, Therrien SC. 2020. Hearing in birds: what changes from air to water. In: Popper AN, Hawkins A, editors. The effects of noise on aquatic life. Springer: New York; 2020. pp. 77–82.

[50] Basan F, Fischer JG, Kühnel D. Soundscapes in the German Baltic Sea before and during the COVID-19 Pandemic. Front Mar Sci. 2021;8:689860.

[51] Bertucci F, Lecchini D, Greeven C, Brooker RM, Minier L, Cordonnier S, et al. Changes to an urban marina soundscape associated with COVID-19 lockdown in Guadeloupe. Environ Pollut. 2021;289:117898. doi: 10.1016/j.envpol.2021.117898 34375848 PMC9188413

[52] March D, Metcalfe K, Tintoré J, Godley BJ. Tracking the global reduction of marine traffic during the COVID-19 pandemic. Nat Commun. 2021 Apr 27;12(1):1–2. 33907197 10.1038/s41467-021-22423-6 PMC8079689

[53] Lincoln FC. The treatment of oil-soaked birds. Wildlife Leaflet 221. US Geological Survey; 1942.

[54] Erickson RC. 1962. Effects of oil pollution on migratory birds. In: Tarzwell CM, editor. Biological Problems in Water Pollution, Third Seminar. Cincinnati: US Department of Health, Education, and Welfare; 1962. pp. 177–181.

[55] Marcella TK, Gende SM, Roby DD, Allignol A. Disturbance of a rare seabird by ship-based tourism in a marine protected area. PLoS One. 2017;12(5):e0176176. doi: 10.1371/journal.pone.0176176 28489902 PMC5425178

[56] Kanjir U, Greidanus H, Oštir K. Vessel detection and classification from spaceborne optical images: A literature survey. Remote Sens. Environ. 2018;207: 1–26. doi: 10.1016/j.rse.2017.12.033 29622842 PMC5877374

[57] Fliessbach KL, Borkenhagen K, Guse N, Markones N, Schwemmer P, Garthe S. A ship traffic disturbance vulnerability index for Northwest European seabirds as a tool for marine spatial planning. Front Mar Sci. 2019;6:192.

